# Association of serum‐based mitophagy biomarkers and spatial navigation performance in Alzheimer´s disease

**DOI:** 10.1002/alz.085847

**Published:** 2025-01-09

**Authors:** Martina Laczó, Katerina Veverova, Zuzana Svacova, Alzbeta Katonova, Evandro Fei Fang, Jakub Hort, Martin Vyhnalek, Jan Laczó

**Affiliations:** ^1^ Memory Clinic, Department of Neurology, Charles University, Second Faculty of Medicine and Motol University Hospital, Prague Czech Republic; ^2^ Memory Clinic, Department of Neurology, 2nd Faculty of Medicine, Charles University and Motol University Hospital, Prague Czech Republic; ^3^ Department of Clinical Molecular Biology, University of Oslo and Akershus University Hospital, Norway, Oslo Norway

## Abstract

**Background:**

Mitophagy is a process of intracellular protein homeostasis through which cells eliminate senescent and dysfunctional mitochondria. Altered mitophagy contributes to Alzheimer´s disease (AD) pathology and is associated with worse cognitive functions. We evaluated association of levels of mitophagy proteins (ULK1, BNIP3L, PINK1, and TFEB in serum and ULK1 and PINK 1 in cerebrospinal fluid [CSF]) with spatial egocentric (body‐centered) and allocentric (world‐centered) navigation performance, which is typically impaired in early stages of AD.

**Method:**

Participants with AD aMCI (n=39), mild AD dementia (n=19), and cognitively normal (CN) older adults (n=20) underwent cognitive assessment and brain MRI. Diagnosis of AD was supported by positivity of AD biomarkers in CSF or amyloid PET imaging. Spatial navigation assessment was performed using real‐space and computerized versions of a human analog of the Morris Water Mate Test (hMWM). The hMWM had four tasks: first, with start position and landmarks (combined allocentric‐egocentric), second, only with start position (egocentric), third, only with landmarks (allocentric), and fourth, with landmarks after 30 minutes (allocentric delayed).

**Result:**

Levels of mitophagy proteins in the CN group were not associated with performance in any of the spatial navigation tasks (ps > .091). In the AD aMCI group, higher levels of CSF PINK1 were associated with more accurate spatial navigation performance in the computerized egocentric‐allocentric task (r = ‐0.39; p = .030) and the association of higher levels of CSF ULK1 with worse spatial navigation performance in computerized allocentric delayed task approached statistical significance (r = 0.36; p = .054). Levels of mitophagy proteins in the AD dementia group were not associated with performance in any of the spatial navigation tasks (ps > .068).

**Conclusion:**

Higher level of CSF PINK1 was associated with more accurate egocentric‐allocentric spatial navigation performance in computerized task and higher levels of CSF ULK were associated with worse allocentric navigation performance. These findings support the hypothesis that normal cognition requires efficient mitophagy and more research is needed to evaluate the role of various mitophagy markers in AD.

